# Genetic inhibition of glutamate allosteric potentiation of GABA_A_Rs in mice results in hyperexcitability, leading to neurobehavioral abnormalities

**DOI:** 10.1002/mco2.235

**Published:** 2023-04-24

**Authors:** Yehong Du, Junjie Li, Maoju Wang, Qiuyun Tian, Yayan Pang, Ya Wen, Dongchuan Wu, Yu Tian Wang, Zhifang Dong

**Affiliations:** ^1^ Growth, Development, and Mental Health of Children and Adolescence Center Pediatric Research Institute Ministry of Education Key Laboratory of Child Development and Disorders National Clinical Research Center for Child Health and Disorders China International Science and Technology Cooperation Base of Child Development and Critical Disorders Chongqing Key Laboratory of Translational Medical Research in Cognitive Development and Learning and Memory Disorders Children's Hospital of Chongqing Medical University Chongqing China; ^2^ Brain Research Centre and Department of Medicine Vancouver Coastal Health Research Institute University of British Columbia Vancouver British Columbia Canada; ^3^ Translational Medicine Research Center China Medical University Hospital Graduate Institutes of Biomedical Sciences Taichung China; ^4^ Institute for Brain Science and Disease of Chongqing Medical University Chongqing China

**Keywords:** epilepsy, excitation‐inhibition balance, GABA_A_ receptor, synaptic transmission

## Abstract

The imbalance between neuronal excitation and inhibition (E/I) in neural circuit has been considered to be at the root of numerous brain disorders. We recently reported a novel feedback crosstalk between the excitatory neurotransmitter glutamate and inhibitory γ‐aminobutyric acid type A receptor (GABA_A_R)‐glutamate allosteric potentiation of GABA_A_R functions through a direct binding of glutamate to the GABA_A_R itself. Here, we investigated the physiological significance and pathological implications of this cross‐talk by generating the β3_E182G_ knock‐in (KI) mice. We found that β3_E182G_ KI, while had little effect on basal GABA_A_R‐mediated synaptic transmission, significantly reduced glutamate potentiation of GABA_A_R‐mediated responses. These KI mice displayed lower thresholds for noxious stimuli, higher susceptibility to seizures and enhanced hippocampus‐related learning and memory. Additionally, the KI mice exhibited impaired social interactions and decreased anxiety‐like behaviors. Importantly, hippocampal overexpression of wild‐type β3‐containing GABA_A_Rs was sufficient to rescue the deficits of glutamate potentiation of GABA_A_R‐mediated responses, hippocampus‐related behavioral abnormalities of increased epileptic susceptibility, and impaired social interactions. Our data indicate that the novel crosstalk among excitatory glutamate and inhibitory GABA_A_R functions as a homeostatic mechanism in fine‐tuning neuronal E/I balance, thereby playing an essential role in ensuring normal brain functioning.

## Graphical abstract

The β3_E182G_ single mutation is sufficient to eliminate the glutamate potentiation of GABA_A_R responses, and to cause electrophysiological and behavioral abnormalities, whereas bilateral hippocampal overexpressions of wild type β3‐containing GABA_A_Rs rescue these abnormalities. We therefore revealed that glutamate‐GABA_A_R crosstalk plays a homeostatic role in maintaining a proper balance between neuronal excitation and inhibition under both physiological and pathological conditions.

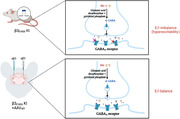

## 1 INTRODUCTION

Neuronal excitation‐inhibition (E/I) balance is fundamental for all aspects of brain functions. Disrupting the balance is believed to be at the root of pathogenesis of neurological illnesses including acute brain dysfunctions like stroke and epilepsy, chronic neurodegenerative diseases like Alzheimer's disease, and mental disturbances like schizophrenia and autism.^1^, ^2^, ^3^, ^4^, ^5^ In the mammalian central nervous system, synaptic excitation is largely mediated by the principal excitatory transmitter glutamate acting at ionotropic glutamate receptors; whereas synaptic inhibition is largely mediated by inhibitory transmitter γ‑aminobutyric acid (GABA) acting on ionotropic GABA A receptor (GABA_A_R). Understanding the mechanisms that control neuronal balance between glutamate excitation and GABA inhibition is always an extensively researched subject in neuroscience, with scientific and clinical significance.

The GABA_A_R is a ligand‐gated heteropentameric chloride ion channel receptor that is assembled from several families of subunits, including α1‐6, β1‐3, γ1‐3, δ, ε, θ, π, and ρ1‐3.^6^, ^7^, ^8^, ^9^ Most GABA_A_Rs are assembled from two α subunits, two β subunits, and one γ subunit.^10^ In our recent report,^11^ using a combination of ligand binding assays, site‐directed mutations, and electrophysiological characterizations in human embryonic kidney (HEK) cells overexpressing recombinant GABA_A_Rs, we identified a novel glutamate binding site in the GABA_A_R at a pocket located in the interface of α^+^ and β^−^ subunits of the receptor and upon binding, glutamate allosterically potentiates the function of the GABA_A_R. Given that this glutamate‐mediated allosteric potentiation of GABA_A_Rs could be demonstrated in primary neurons, as evidenced by glutamate potentiation of both inhibitory postsynaptic currents (IPSCs) and inhibitory tonic currents, we hypothesized that this novel glutamate‐GABA_A_R crosstalk, that is, the allosteric potentiation of GABA_A_R by glutamate, may function as an essential homeostatic feedback mechanism of fine‐tuning neuronal E/I balance, thereby having major physiological and/or pathological consequences. Indeed, mice with β2 E181G GABA_A_R subunit knock‐in (KI) inhibited glutamate potentiation of GABA_A_R activity while maintaining baseline GABAAR‐mediated synaptic currents, and this mouse line had abnormal phenotypes of sensory process, as well as social interactions; and a pathological condition exhibiting increased kainic acid (KA)‐induced seizure activity.^11^

The presence of a β subunit is critical for the formation of functional native GABA_A_Rs. Importantly, the critical pocket‐forming glutamic acid residue is conserved among all β subunits (E182 in β2 and β3, and E181 in β1) and single mutation of this residue into alanine almost abolishes glutamate modulation without affecting normal GABA_A_R function.^7^ The β3 subunit is more broadly expressed than the β2 subunit in the mammalian brain, including cerebral cortex, hippocampus and hypothalamus,^12^, ^13^ and serves as one of the most important subunits regulating GABA_A_R function at both circuit^14^ and behavioral levels.^1^, ^2^, ^15^, ^16^ Therefore, we hypothesized that mice harboring glutamate‐binding deficient GABA_A_Rs generated by KI of β3 Glutamic acid 182 to Glycine (E182G) mutation would have significantly diminished glutamate potentiation on most native GABA_A_Rs, thereby exhibiting overexcitation phenotypes.

Here, our findings reveal that β3_E182G_ single point mutation is adequate to largely eliminate the glutamate potentiation of GABA_A_R responses, and to cause electrophysiological and behavioral abnormalities. More importantly, we also report that bilateral hippocampal wild type β3‐containing GABA_A_Rs overexpression is sufficient to rescue the deficits of glutamate potentiation of GABA_A_R‐regulated responses along with hippocampus‐related behavioral abnormalities. The present study not only provides additional support for a newly identified glutamate allosteric potentiation of GABA_A_Rs but most importantly demonstrates its importance as a physiological and pathophysiological mechanism by which neuronal E/I balance is controlled in mammalian brains.

## 2 RESULTS

### 2.1 β3_E182G_ mutation impairs glutamate potentiation of GABA_A_R‐mediated responses

**FIGURE 1:**
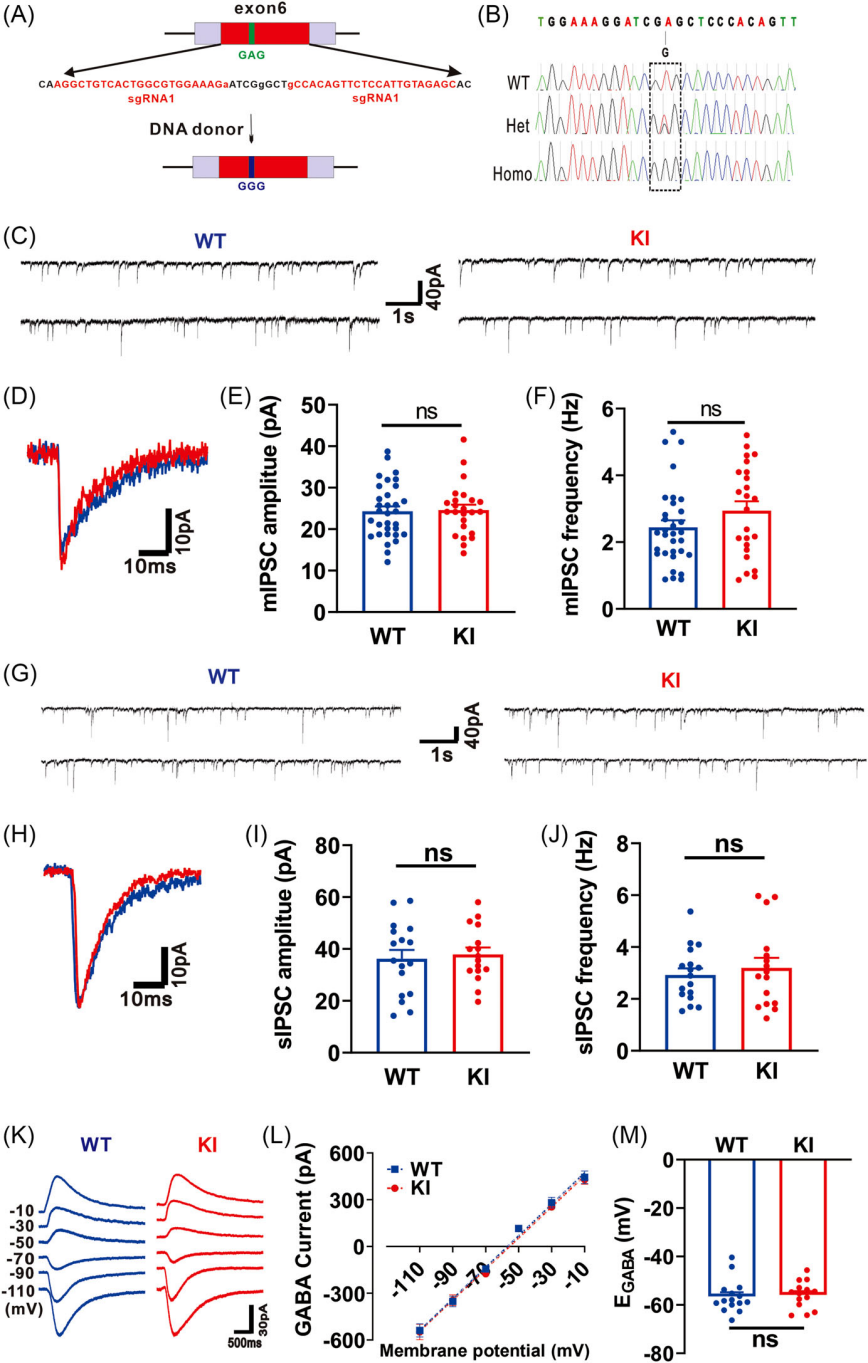
Generation and electrophysiological characterization of β3_E182G_ knock‐in (KI) mice. (A and B) DNA genotyping confirms the correct GAG‐GGG mutation that converts glutamic acid at 182 residue of β3 subunit of WT mice into glycine (β3_E182G_) in both heterozygous (Het) and homozygous (Homo) mice. (C–J) Whole‐cell voltage‐clamp recordings of hippocampal CA1 neurons in slices reveal no significant alteration in GABA_A_R‐mediated miniature inhibitory postsynaptic currents (mIPSCs) and spontaneous GABAergic inhibitory currents (sIPSCs) in β3_E182G_ KI mice in comparison with WT mice. Representative traces of continuous mIPSCs are shown in (C) and individual traces of mIPSCs from WT in blue and from KI in red are shown in (D). Bar graphs of the amplitude (E) and frequency (F) of mIPSCs show no significant difference between neurons from WT (blue bars; *n* = 32) and from KI (red bars; *n* = 24) mice. Representative traces of continuous sIPSCs are shown in (G) and individual traces of mIPSCs from WT in blue and from KI in red are shown in (H). Bar graphs of the amplitude (I) and frequency (J) of sIPSCs show no significant difference between neurons from WT (blue bars; *n* = 17) and from KI (red bars; *n* = 16) mice. (K–M) Gramicidin‐perforated patch‐clamp recordings of hippocampal CA1 neurons in slices reveal no significant alteration in GABA reversal potential. Representative traces are shown in (K) and individual graph of I–V plots for the traces from WT in blue and from KI in red are shown in (L). Quantification of E_GABA_ (M) reveals no significant difference between neurons from WT (blue bars; *n* = 15) and from KI (red bars; *n* = 14). Data are expressed as mean ± SEM; ns, no significant difference.

To examine the physiological and pathological roles of this glutamate‐GABA_A_R crosstalk in intact animals, we generated KI mice with wild type (WT) endogenous β3 subunits replaced with mutated β3_E182G_ subunits (Figure 1A). This mutation changed codon 182 from guanine‐adenine‐guanine (GAG) to guanine‐guanine‐guanine (GGG), resulting in the amino acid change of glutamic acid to glycine (E182G) of β3. Then deoxyribonucleic acid (DNA) genotyping was used to confirm the successful production of β3_E182G_‐KI (Figure 1B). We found that homozygous KI mice were fully fertile, along with a slightly slower growth rate (homozygous KI mice were approximately 10% lighter compared to WT mice) (Figure S1).

To electrophysiologically characterize the impact of the mutation on GABA_A_R function, we performed whole‐cell patch‐clamp recordings of hippocampal CA1 cells from both homozygous KI and WT littermate mice at the age of postnatal day 90. We first determined if the mutation would affect basal GABA_A_R function through recordings of pharmacologically isolated miniature IPSCs (mIPSCs) and spontaneous inhibitory post‐synaptic current (sIPSCs) mediated by GABA_A_Rs. The results showed that the mutation had no effect on basal mIPSCs and sIPSCs, as reflected by no alterations of their amplitude (for mIPSCs: WT: 24.31 ± 1.17pA, n = 32; KI: 24.62 ± 1.28pA, *n* = 24; *p* = 0.860; Figure 1C‐‐E; for sIPSCs: WT: 36.23 ± 3.39pA, *n* = 17; KI: 37.89 ± 2.67pA, *n* = 16; *p* = 0.705; Figure 1G‐‐I) and frequency (for mIPSCs: WT: 2.44 ± 0.21 Hz, *n* = 32; KI: 2.94 ± 0.28 Hz, *n* = 24; *p* = 0.155; Figure 1F; for sIPSCs: WT: 2.92 ± 0.25 Hz, *n* = 17; KI: 3.19 ± 0.39 Hz, *n* = 16; *p* = 0.557; Figure 1G) compared with WT. To directly test the changes in chloride homeostasis of hippocampal CA1 cells, the GABA reversal potential (E_GABA_) was measured. The results showed that there was no significant difference in E_GABA_ between WT (−56.49 ± 1.74, *n* = 15; Figure 1K‐‐M ) and KI (−55.84 ± 1.46, *n* = 14; *p* = 0.782; Figure 1K‐‐M ) mice. Thus, consistent with the observations made with the mutation in the recombinant expression system,^11^ knocking in the single E182G at the β3 subunit appeared to have no significant impact on the basal function of synaptic GABA_A_Rs.

**FIGURE 2:**
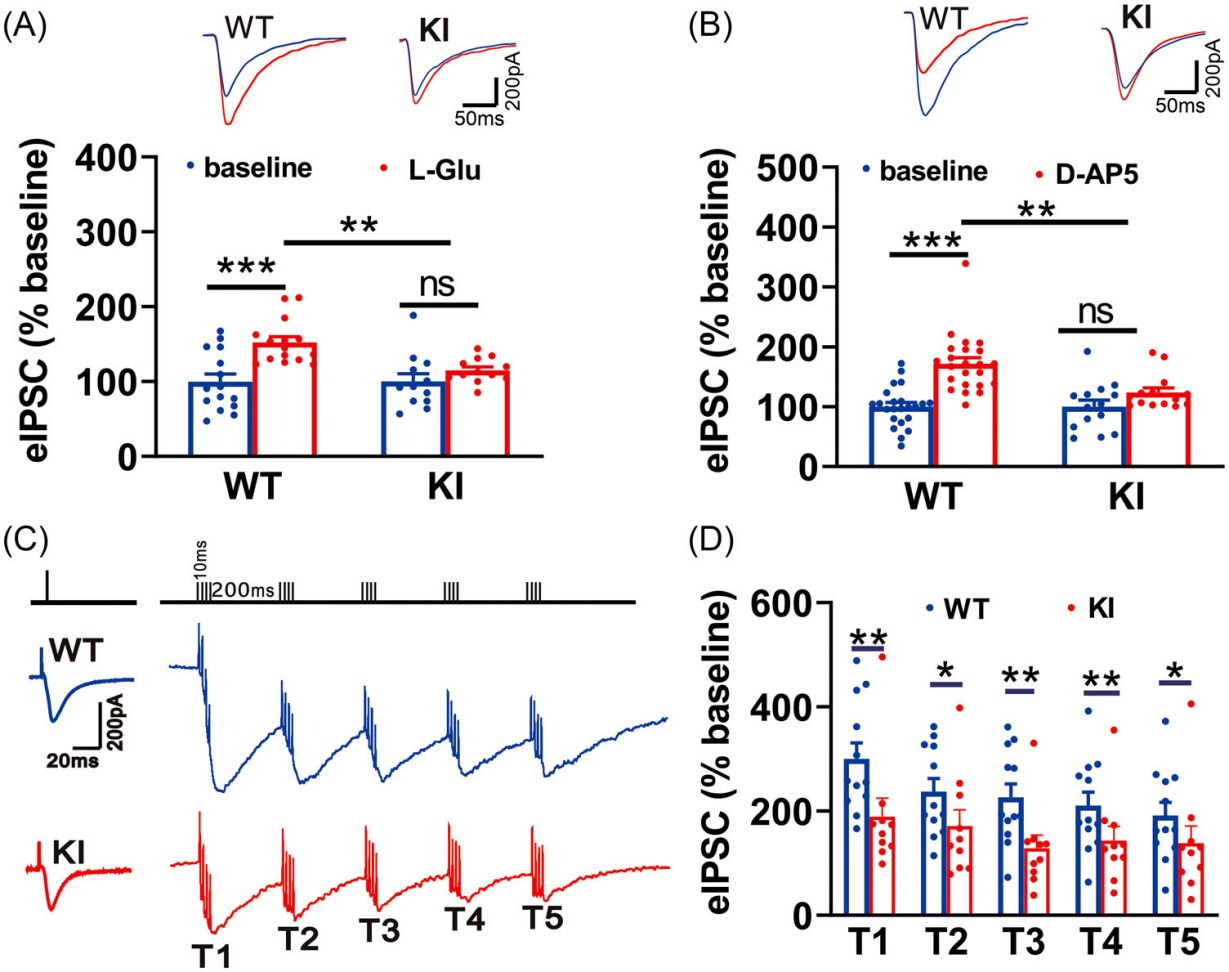
Glutamate‐GABA_A_R cross‐talk is impaired in β3_E182G_ knock‐in (KI) mice. (A and B) Bath application of L‐glutamate (A; L‐Glu, 50 μM; WT, *n* = 15; KI, *n* = 12) or D‐AP5 (B; D‐AP5, 50 μM; WT, *n* = 23; KI, *n* = 14) potentiates inhibitory postsynaptic currents (IPSCs) only in slices from WT, but not in slices from KI mice. (C and D) Potentiation of IPSCs by theta‐burst stimulation (TBS; top panel in C) is significantly reduced in slices from KI mice (representative traces in C and bars in D; *n* = 9) in comparison with that in slices from WT mice (representative traces in C and bars in D; *n* = 12). The amplitudes of IPSCs evoked by TBS are normalized to their own IPSC evoked with a single pulse. Data are expressed as mean ± SEM; *p < 0.05; **p < 0.01; ***p < 0.001.

We then tested whether β3_E182G_ KI affects glutamate‐mediated allosteric potentiation of GABA_A_R activity by comparing effects of exogenously applied L‐glutamate (L‐Glu) or D‐AP5, an N‐methyl‐D‐aspartate (NMDA) receptor antagonist, on electrical stimulation‐evoked GABA_A_R‐mediated IPSCs (eIPSCs). We found that bath application of L‐Glu (50 μM) significantly potentiated GABA_A_R‐mediated IPSCs in WT mouse brain slices (WT: 152.13 ± 5.92% relative to baseline, *n* = 15; *p* < 0.001; Figure 2A) but could not alter eIPSCs in KI mouse brain slices (KI: 115.01 ± 4.72% relative to baseline, *n* = 12; *p* = 0.206, KI vs. baseline; *p* = 0.001, KI vs. WT; Figure 2A). We substituted L‐Glu with D‐AP5, which has been demonstrated a potent agonist of the glutamate‐binding site on GABAARs, to exclude the potential requirement of glutamate receptor activation in glutamate‐induced potentiation.^11^ These results showed that D‐AP5 (50 μM) reliably potentiated GABA_A_R IPSCs in WT mouse slices (WT: 171.58% ± 10.03% relative to their own baseline, *n* = 23; *p* = 0.001; Figure 2B) but could not enhance eIPSCs in KI mouse slices (KI: 123.77% ± 7.74% relative to baseline, *n* = 14; *p* = 0.085, KI vs. baseline; *p* = 0.002, KI vs. WT; Figure 2B). To examine the effect of endogenous glutamate on the allosteric potentiation of GABA_A_Rs under certain physiological conditions in WT and KI mice, a 5 trains of 4 pulses of theta‐burst stimulus (TBS; Figure 2C) was provided to enhance the release of endogenous glutamate from presynaptic glutamate terminals and to compare its implications on pharmacologically isolated eIPSCs (following the blockage of AMPAR‐ and NMDAR‐mediated EPSCs). These results demonstrated that the summation amplitude of IPSCs produced by TBS was considerably larger in WT mouse sections than in KI mouse sections (*p* < 0.02–0.001 for T1‐T5; Figure 2D). The TBS induced amplitudes of IPSCs were 300.16% ± 30.81%, 237.50% ± 24.86%, 226.45% ± 26.01%, 210.43% ± 25.93%, and 191.43% ± 25.47% of the baseline for train (T) 1–5 in WT mouse brain slices (WT: T1‐T5, *n* = 12; Figure 2D); whereas the amplitudes were 155.11% ± 11.78%, 146.07% ± 20.52%, 106.48% ± 12.44%, 120.17% ± 14.93%, and 108.73% ± 15.65% for T1–T5 in KI mouse brain slices (KI: T1‐T5, *n* = 10; Figure 2D). The results strongly support our assumption that increased concentration of endogenous glutamate during TBS stimulation is sufficient to engage the glutamate‐GABA_A_R crosstalk to potentiate the native GABA_A_Rs, thereby playing a negative feedback role in fine‐tuning neuronal E‐I balance in the brain.

### 2.2 β3_E182G_ KI mice display enhanced learning and memory

**FIGURE 3:**
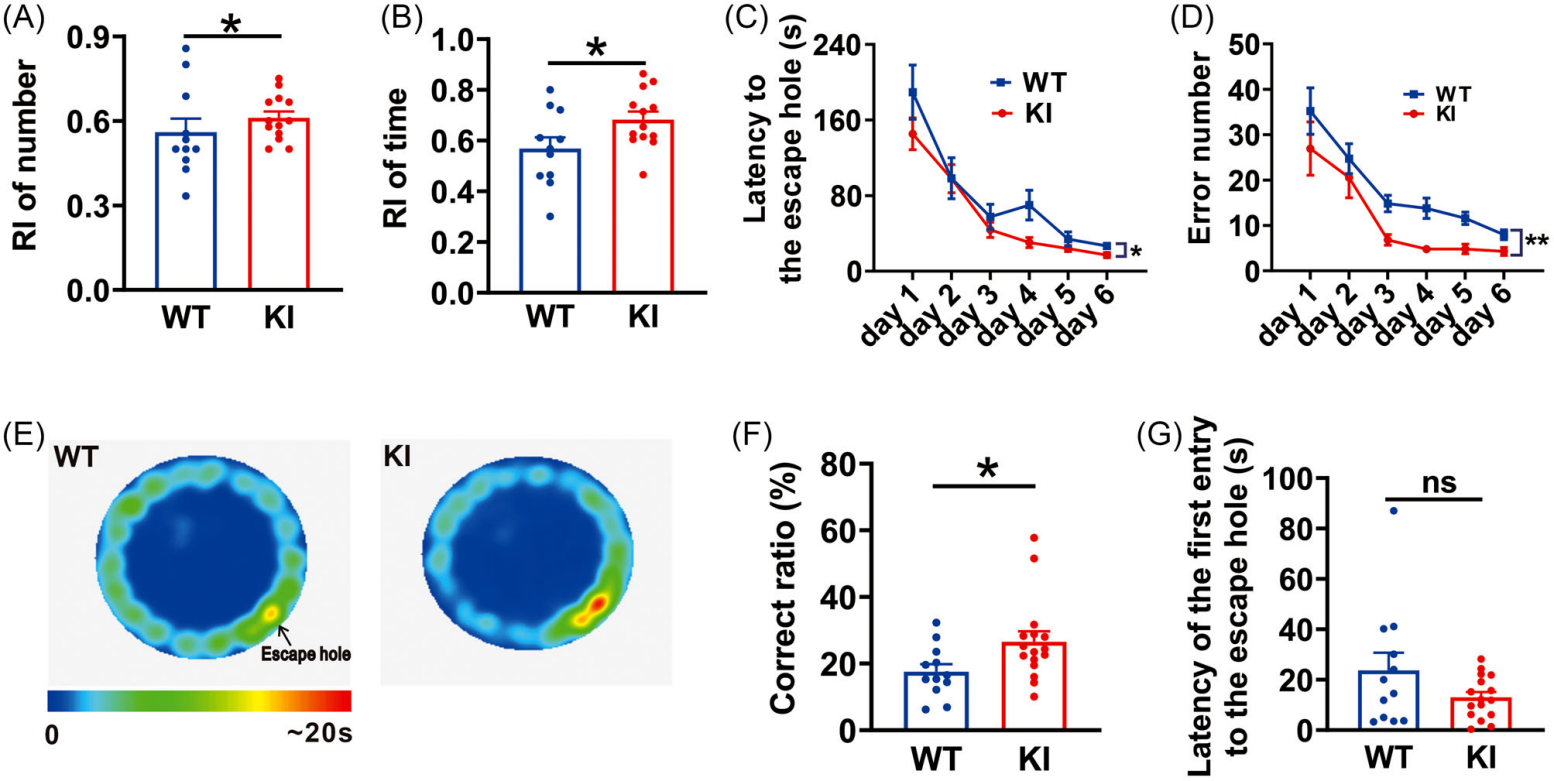
β3_E182G_ knock‐in (KI) mice exhibit enhanced learning and memory in both novel object recognition and Barnes maze tests. (A and B) In novel object recognition test, both number (A) and time (B) of the recognition index (RI) are increased in KI mice (KI; *n* = 13), compared with WT mice (WT; *n* = 11). (C–G) In the Barnes maze test, both latency (C) and error number (D) to find the escape hole are significantly decreased in KI mice (KI; *n* = 16), compared with WT mice (WT; *n* = 15) during learning phase. During the probe phase, the correct ratio (E and F) is significantly increased in KI mice, compared with WT mice, and the latency to the first entry to the escape hole (G) shows a trend of decrease. Data are expressed as mean ± SEM; *p < 0.05; **p < 0.01; ***p < 0.001.

The above observations in *in*‐*vitro* slice preparations confirm a significant reduction in glutamate allosteric potentiation of GABA_A_R function in KI‐β3_E182G_ mice. Compromising glutamate‐GABA_A_R negative feedback mechanism may lead to behavioral abnormalities in these KI mice. We first examined if there was any alteration in cognitive abilities of KI mice using novel object recognition task and Barnes maze assessments (Figure 3). In the novel objective recognition test, we found that both recognition index (RI) number (WT: 0.56 ± 0.04, *n* = 11; KI: 0.64 ± 0.02, *n* = 13; *p* = 0.038; Figure 3A) and time (WT: 0.57 ± 0.04, *n* = 11; KI: 0.68 ± 0.03, *n* = 13; *p* = 0.019; Figure 3B) were significantly increased in KI mice compared to WT controls, suggesting an enhanced object recognition in KI mice. Similar results were also observed in Barnes maze test (Figure 3C–G). During the acquisition phase, KI mice (*n* = 16), compared with WT control (*n* = 12), showed significantly shorter escape latencies (*p* = 0.044, KI vs. WT; Figure 3C) and smaller error numbers (*p* = 0.005, KI vs. WT; Figure 3D) to find the escape hole. On memory retrieval test, the KI mice showed significantly better performance than the WT controls as evidenced by the increased correct ratio to find the escape hole (WT: 17.57 ± 2.24%, *n* = 12; KI: 26.59 ± 3.09%, *n* = 16; *p* = 0.036; Figure 3E,F), although there was no group difference in latency to find the escape hole for the first time (WT: 23.69 ± 6.98s, *n* = 12; KI: 13.01 ± 2.11s, *n* = 16; *p* = 0.091; Figure 3G). Taken together, these findings indicate that the performance of cognitive abilities assessed by the two tests is not impaired but enhanced in KI mice.

### 2.3 β3_E182G_ KI mice exhibit higher neuronal excitability characteristics

**FIGURE 4:**
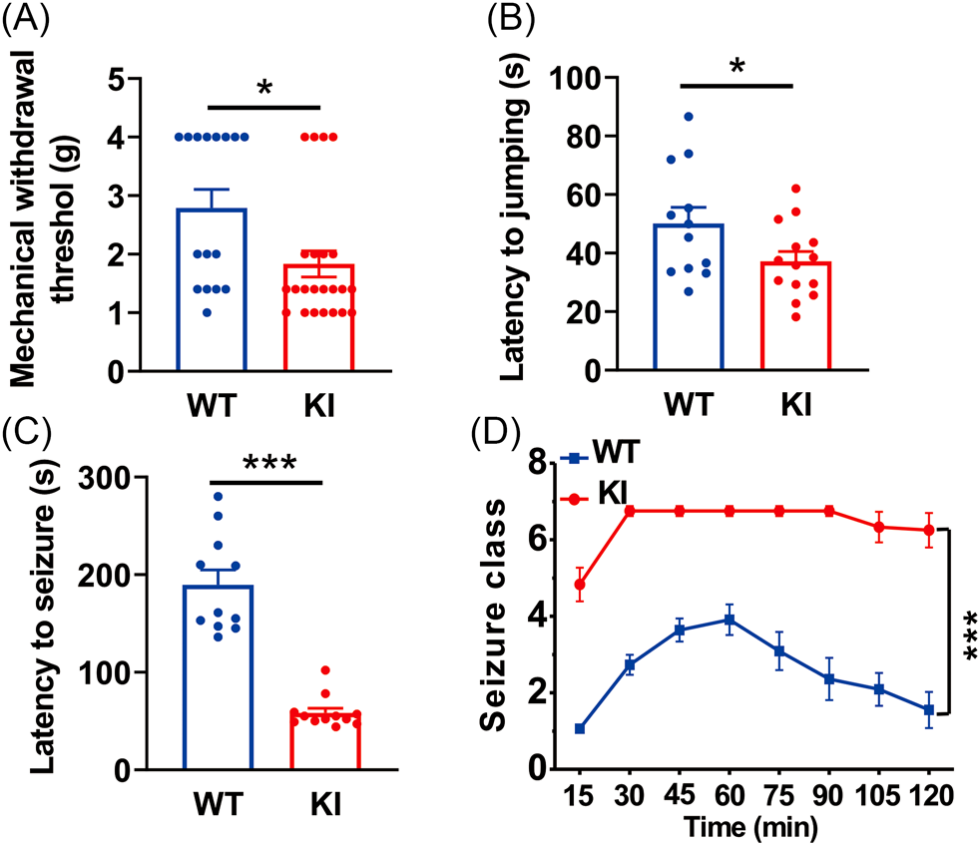
β3_E182G_ knock‐in (KI) mice exhibit neuronal overexcitation‐related behavioral phenotypes. (A and B) Homozygous KI mice have significantly decreased thresholds to both pressure (A; WT, *n* = 16; KI, *n* = 23) and temperature (B; WT, *n* = 12; KI, *n* = 14) stimuli to the limbs. (C and D) Homozygous KI mice exhibit increased susceptibility to kainic acid (KA; 20 mg/kg; i.p.)‐induced seizure activity. Compared with WT mice (WT; *n* = 11), KI mice (KI; *n* = 12) had significantly decreased latency (C) and increased severity (D) of the KA‐induced seizure activity. Data are expressed as mean ± SEM; *p < 0.05, ***p < 0.001.

By disrupting glutamate‐GABA_A_R crosstalk‐mediated negative feedback in controlling neuronal excitability, the β3_E182G_ KI mice are expected to exhibit certain neuronal overexcitation phenotypes. As predicted, in comparison with WT mice, KI mice showed neuronal hyper‐excitability, as evidenced by a reduced threshold for noxious mechanical (WT: 2.79 ± 0.32 g, *n* = 16; KI: 1.83 ± 0.22 g, *n* = 23; *p* = 0.021; Figure 4A) and temperature stimulations (WT: 50.12 ± 5.47s, *n* = 12; KI: 37.23 ± 3.35s, *n* = 14; *p* = 0.049; Figure 4B). Then the well‐characterized KA (20 mg/kg; i.p.) mouse model of epilepsy was introduced to further corroborate the higher neuronal network excitability characteristics of the KI mice. These findings revealed that in comparison to WT mice KA‐induced seizure activity was significantly increased in KI mice, as evidenced by reduced latency (WT: 189.64 ± 15.25s, *n* = 11; KI: 58.33 ± 4.67s, *n* = 12; *p* < 0.001; Figure 4C) and increased severity (*p* < 0.001; Figure 4D) of KA‐induced seizure activity. These results not only further support that the KI mice have a deficit in glutamate potentiation of GABA_A_R function but also provide additional evidence for a critical negative feedback role of this modulation in controlling neuronal E/I balance.

### 2.4 β3_E182G_ KI mice display autistic‐like behaviors

**FIGURE 5:**
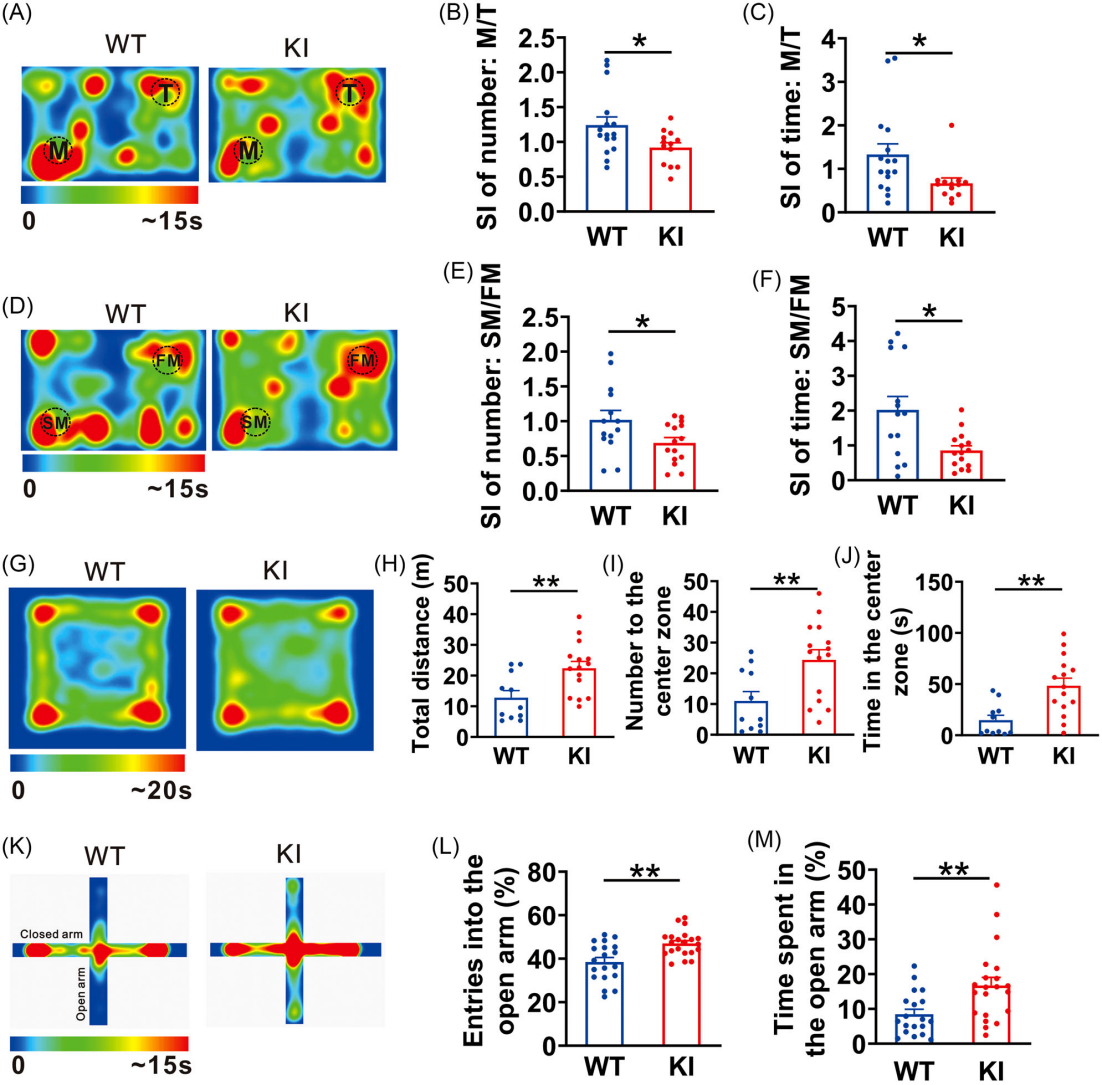
β3_E182G_ knock‐in (KI) mice exhibit atypical autistic‐like behaviors. (A–F) Social interaction in KI mice is significantly impaired, during test 1 of toy (T) verse mouse (M) (A), both number (B) and time (C) of the social index (SI) are significantly decreased in KI mice (KI; n = 13), compared with WT mice (WT; n = 14). During the test when the toy was replaced by another stranger mouse (SM), the other one was a familiar mouse (FM) (D), the KI mice also show impaired SI number (E) and time (F), compared with WT mice. (G–M) KI mice exhibit increased locomotor activity and reduced anxiety‐like behaviors. In the open field test (G), the spontaneous traveling distance (H), the number (I) of entry into the central zone and the time (J) spent in the central zone were significantly increased in KI mice (KI; *n* = 15), compared with that of WT mice (WT; *n* = 11). In elevated plus maze test (K), the entries (L) into the open arms and time (M) spent in the open arms are significantly increased in KI mice (KI; *n* = 21), compared with that in WT mice (WT; *n* = 19). Data are expressed as mean ± SEM; *p < 0.05; **p < 0.01; ***p < 0.001.

Previous studies have revealed that the disruption of E/I balance contributes to the pathogenesis of autism spectrum disorders (ASD).^16^, ^17^, ^18^, ^19^, ^20^ We, therefore, investigated if an alteration of autistic‐like behaviors occurred in the β3_E182G_ KI mice by evaluating their performances in social interaction and locomotor activity. In a three‐chambered social interaction test with a mouse (M) versus a toy (T), we found that the KI mice displayed obvious social deficits as reflected by a significant reduction in both number (WT: 1.24 ± 0.12, *n* = 16; KI: 0.92 ± 0.07, *n* = 13; *p* = 0.026; Figure 5A,B) and time (WT: 1.33 ± 0.24, *n* = 16; KI: 0.67 ± 0.12, *n* = 13; *p* = 0.024; Figure 5A,C) of the social index (SI), compared to WT controls. Immediately after test, the test mouse was co‐housed with the paired mouse during test for 24 h to familiarize with it (FM). Twenty‐four hours later, the second test was performed to determine the social novelty preference in the KI mice. During this test, we replaced the toy with another age‐ and gender‐matched stranger mouse (SM). These results again showed that compared with WT controls, the KI mice exhibited a markedly reduced SI in both number (WT: 1.02 ± 0.13, *n* = 14; KI: 0.64 ± 0.08, *n* = 15; *p* = 0.026; Figure 5D,E) and time (WT: 2.02 ± 0.38, *n* = 14; KI: 0.75 ± 0.12, *n* = 15; *p* = 0.008; Figure 5D,F). We next evaluated potential changes in locomotor activity using an open‐field test (Figure 5G–J). As we expected, spontaneous locomotor activity in KI mice was significantly increased, as evidenced by the increased total traveling distance during the test (WT: 12.83 ± 2.26 m, *n* = 11; KI: 22.40 ± 2.19 m, *n* = 15; *p* = 0.006; Figure 5G,H). Surprisingly, this increased locomotor activity appeared to be associated with a reduced level of anxiety. The results showed that the KI mice displayed increased numbers of entry into the center zone (WT: 11.00 ± 3.05, *n* = 11; KI: 24.40 ± 3.27, *n* = 15; *p* = 0.008; Figure 5G,I) and  more time (WT: 14.77 ± 4.75s, *n* = 11; KI: 48.47 ± 7.39s, *n* = 15; *p* = 0.002; Figure 5G,J) in the center zone of the testing chamber compared with their WT counterparts. Such reduced anxiety phenotype was not expected as neuronal overexcitation due to disrupted E/I imbalance is often associated with an increased anxiety.^21^ We, therefore, further assessed the alteration of anxiety level in the KI mice using the elevated plus maze test (Figure 5K‐M). Consistent with a reduced level of anxiety, the KI mice showed an increase in both percentage of total numbers of entry into the open arms (WT: 38.49% ± 2.04%, *n* = 19; KI: 47.01% ± 1.29%, *n* = 21; *p* = 0.001; Figure 5K,L) and percentage of total time spent in the open arms (WT: 8.49% ± 1.41%, *n* = 19; KI: 16.72% ± 2.34%, *n* = 21; *p* = 0.006; Figure 5K,M). Thus, the KI mice appear to have atypical ASD phenotypes.

### 2.5 Hippocampal expression of α1β3 reverses the impairments of glutamate allosteric potentiation of GABA_A_Rs in β3_E182G_ KI mice

**FIGURE 6:**
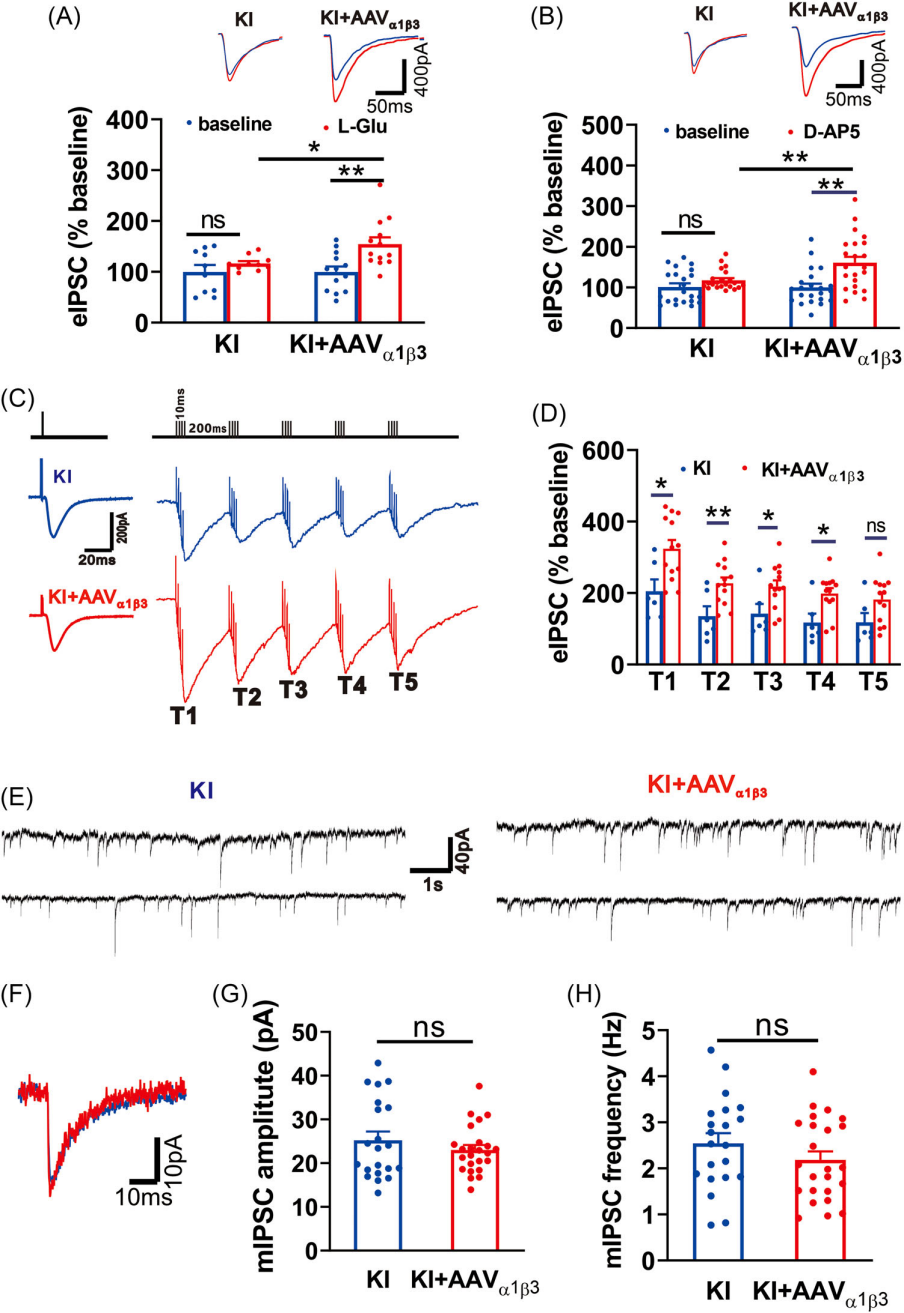
Bilateral expression of wildtype α1/β3 GABA_A_Rs into the hippocampus of β3_E182G_ knock‐in (KI) mice does not alter GABA_A_R‐mediated synaptic transmission, but fully restore glutamate potentiation of receptor‐mediated inhibitory postsynaptic currents (IPSCs). (A–D) The pyramidal neurons coexpressed α1 and β3 subunits of GABA_A_Rs are selected for whole‐cell recordings in slices. The electrical stimulation‐evoked GABA_A_R‐mediated IPSCs are significantly potentiated by bath application of L‐glutamate (A; L‐Glu, 50 μM; KI, *n* = 10; KI+AAV_α1β3_, *n* = 13) or glutamate‐like ligand D‐AP5 (B; D‐AP5, 50 μM; KI, *n* = 22; KI+AAV_α1β3_, *n* = 22) or by TBS (C and D; KI, *n* = 6; KI+AAV_α1β3_, *n* = 13) in hippocampal slices of KI mice virally infected with AAV_α1β3_, but not in slices of non‐infected KI mice. (E–H) Overexpression of α1/β3 subunits of GABA_A_Rs does not alter basal GABA_A_R‐mediated synaptic transmission. Whole‐cell recordings of mIPSCs (E and F) reveal that in comparison with nonvirally infected KI mice (KI), KI mice infected with α1/β3 do not show significant change in either amplitude (G; KI, *n* = 21; KI+AAV_α1β3_, *n* = 24) or frequency (H; KI, *n* = 21; KI+AAV_α1β3_, *n* = 24) of mIPSCs. Data are expressed as mean ± SEM; *p < 0.05; **p < 0.01.

To determine if the impairment of glutamate allosteric modulation of GABA_A_Rs and behavioral changes in the KI mice are results of abnormal development or dynamic alteration of neuronal E/I balance due to the point mutation of residue E182 of β3, we overexpressed functional WT β3 subunit‐containing GABA_A_Rs in hippocampal neurons by bilateral intra‐hippocampal microinjection of adeno‐associated viruses carrying WT β3 (AAV_β3_), and examined its ability to rescue the electrophysiological and behavioral phenotypes in the KI mice. Since topically expressed single GABA_A_R subunit may not efficiently heteromerize with the endogenous GABA_A_R subunits,^22^ we also co‐transfected neurons with the α1 (AAV_α1_) to facilitate the formation of α1 and β3 recombinant receptors (AAV_α1β3_), thereby complementing β3_E182G_‐containing GABA_A_Rs in the KI mice. We first studied the functional rescue of glutamate allosteric potentiation of GABA_A_Rs in hippocampal slices. We recorded the hippocampal CA1 pyramidal neurons that were co‐expressing fluorescently identifiable recombinant GABA_A_Rs containing both α1 (Green) and β3 (Red) (Figure S2). These results showed that overexpressing α1β3 recombinant GABA_A_Rs fully restored the glutamate‐mediated allosteric potentiation of GABA_A_R function (Figure 6A,D), as evidenced by the significant potentiation of GABA_A_R‐mediated whole‐cell currents by bath application of L‐Glu (KI+AAV_α1β3_; 50 μM; *n* = 13; Figure 6A) or D‐AP5 (KI+AAV_α1β3_; 50 μM; *n* = 22; Figure 6B), and GABA_A_R‐mediated IPSCs by TBS (KI+AAV_α1β3_; T1‐T5; *n* = 13; Figure 6C,D). The functional rescue appeared to be due to the expression of WT β3‐containing GABA_A_Rs that enabled the glutamate‐GABA_A_R feedback cross‐talk, and not a result of simply increased numbers of GABA_A_R, since there was no significant difference in amplitude, frequency of mIPSPs between neurons infected with (KI+AAV_α1β3_; Figure 6E,H) and without (KI; Figure 6E,H) AAV_α1β3_ recombinant GABA_A_Rs. These results indicate that overexpressing WT β3 subunit‐containing GABA_A_Rs in the KI mice is sufficient to rescue the deficient glutamate allosteric potentiation of GABA_A_R‐mediated neuronal inhibition without affecting the receptor‐mediated basal synaptic transmission.

### 2.6 Hippocampal expression of α1β3 subunits of GABA_A_Rs rescues behavioral abnormalities in β3_E182G_ KI mice

**FIGURE 7:**
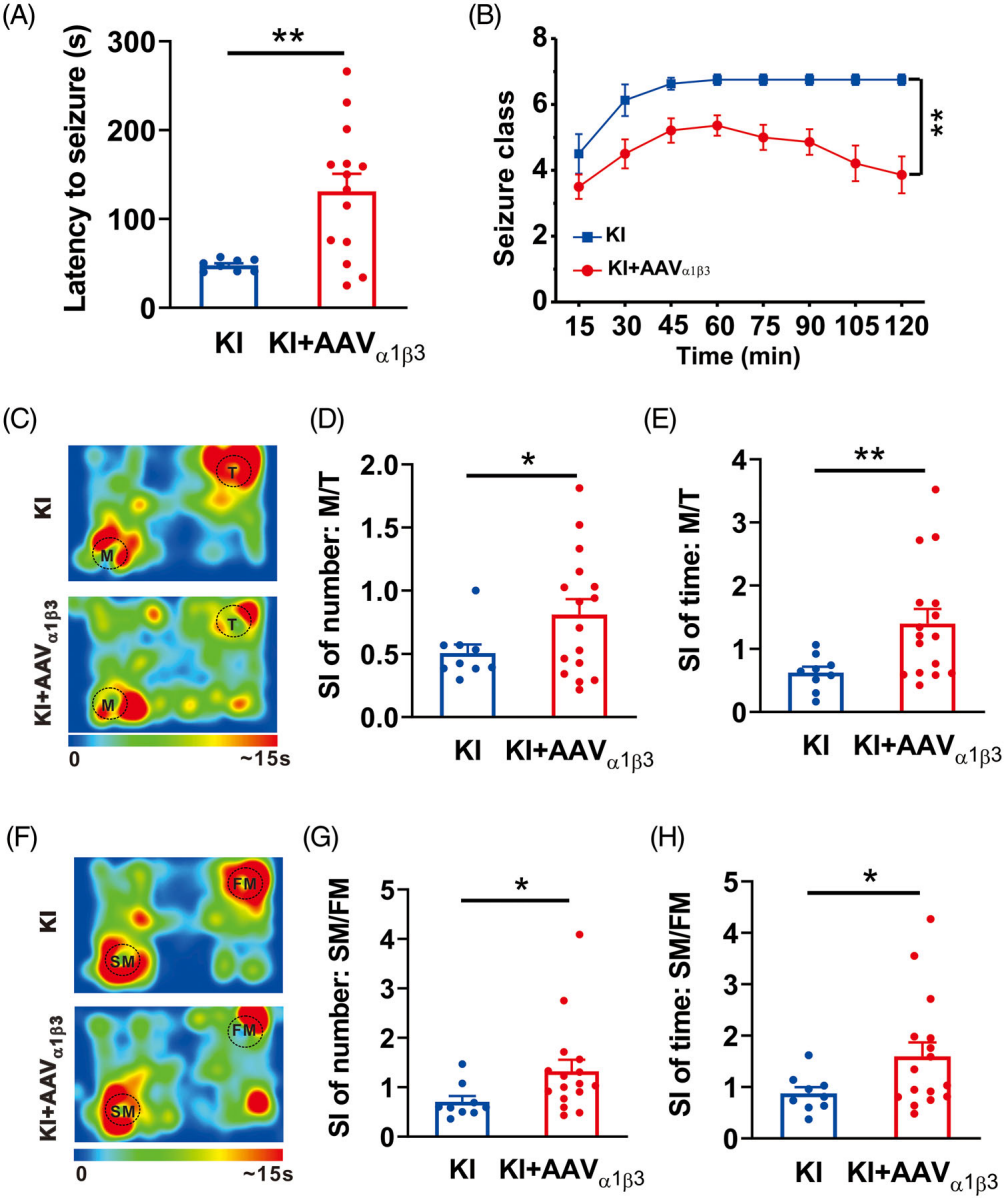
Bilateral expression of wildtype α1/β3 GABA_A_Rs into the hippocampus of β3_E182G_ knock‐in (KI) mice restores hippocampus‐related behavioral phenotypes. (A and B) Hippocampal infection of AAV_α1β3_ into KI mice (KI+AAV_α1β3_; *n* = 14), in comparison with noninfected KI mice (KI; *n* = 8), increased seizure latency (A), and decreased severity (B) of the seizure activity induced by KA (20 mg/kg, i.p.).(C–H) Overexpression of α1/β3 subunits of GABA_A_Rs in the hippocampus of KI mice restores the impairment of social interaction. During test 1 of toy (T) versus mouse (M) (C), both the social index (SI) number (D) and time (E) are increased in KI mice infected with AAV_α1β3_ (KI+AAV_α1β3_; *n* = 16), compared with KI mice (KI; *n* = 9). When the toy was replaced by another stranger mouse (SM), and the other was a familiar mouse (FM) on test 2 (F), the KI mice transfected with AAV_α1β3_ (KI+AAV_α1β3_; *n* = 16) also display improved SI number (G) and time (H), compared with KI mice (KI; *n* = 9). Data are expressed as mean ± SEM; *p < 0.05; **p < 0.01.

Based on the ability of overexpressing α1β3 subunits of GABA_A_Rs to restore the defective glutamate‐GABA_A_R feedback cross‐talk in the KI mice, we predicted that the bilateral hippocampal infection of AAV_α1β3_ should also be able to rescue some of the hippocampus‐related abnormal phenotypes in the β3_E182G_ KI mice, particularly the increased KA‐induced seizure activity and impaired social interactions. Consistent with our reasoning, we found that overexpression of α1β3 (KI+AVV_α1β3_, *n* = 14; Figure 7A,B) significantly reduced epileptic seizures, compared to KI mice (KI, *n* = 8; Figure 7A,B). There was a significant increase in the latency (KI: 47.88 ± 2.35; *n* = 8; KI+AAV_α1β3_: 131.14 ± 19.62s; *n* = 14; *p* = 0.001; Figure 7B) and decrease in the severity (KI+AAV_α1β3_: *n* = 14; KI: *n* = 8; *p* = 0.001; Figure 7B) of KA‐induced seizure activity in the KI mice infected with AVV_α1β3_. Similarly, we found that the KI overexpressing α1β3 (KI+AVV_α1β3_; *n* = 16; Figure 7C,D), in comparison with the KI counterparts (KI, *n* = 9; Figure 7C,D), showed significantly improved social ability, as evidenced by an increase in SI number (KI: 0.51 ± 0.07; KI+AAV_α1β3_: 0.81 ± 0.12; *p* = 0.039; Figure 7C,D) and time (KI: 0.62 ± 0.09; KI+AAV_α1β3_: 1.40 ± 0.23; *p* = 0.005; Figure 7C,E) in mouse (M) versus toy (T) social interaction test and also in SI number (KI: 0.71 ± 0.12; KI+AAV_α1β3_: 1.32 ± 0.21; *p* = 0.027; Figure 7F,G) and time (KI: 0.88 ± 0.12; KI+AAV_α1β3_: 1.60 ± 0.25; *p* = 0.026; Figure 7F,H) in the stranger (SM) versus familiar mouse (FM) social novelty preference. Taken together, these findings demonstrate that expression of recombinant GABA_A_Rs containing the WT β3 subunits can rescue electrophysiological and behavioral phenotypes due to β3_E182G_ mutation‐induced disruption of glutamate‐GABA_A_R negative feedback cross‐talk. In addition, the results strongly suggest that some of the hippocampus‐related behavioral abnormalities in these KI mice are the results of functional, rather than developmental, alteration of the glutamate‐GABA_A_R feedback loop as they can be rescued at adulthood.

## 3 DISCUSSION

Maintaining a proper neuronal E/I balance is fundamental for brain functioning. Despite intensive studies over the last few decades, detailed mechanisms regulating E/I balance in mammalian brains remain poorly understood. In our recent study,^11^ we have identified a novel glutamate‐binding site on the GABA_A_R, at which glutamate acts to allosterically potentiate GABA_A_R function. The presence of such a functional cross‐talk between the principle excitatory transmitter glutamate and the major inhibitory GABA_A_R predicts a critical homeostatic feedback role of the crosstalk in fine‐tuning neuronal E/I balance. With no specific antagonist to inhibit glutamate from binding to this newly identified site on GABA_A_R, we have examined the potential involvement of this cross‐talk under both physiological and pathological conditions through the generation of a β3_E182G_ KI mouse line in which the glutamate binding pocket in most of the native GABA_A_Rs is largely inhibited. β3_E182G_ mutation is artificial and has not been found in the human brain. We provide direct evidence that genetically impairing this glutamate‐GABA_A_R cross‐talk disrupts neuronal E/I balance, leading to neurophysiological and behavioral phenotypes with characteristics of hyper neuronal excitability. Thus, our work demonstrates that through a homeostatic feedback mechanism, this newly identified glutamate‐GABA_A_R crosstalk plays an indispensable role in fine‐tuning neuronal E/I balance under both physiological and pathological conditions.

The co‐release of glutamate with GABA at GABAergic terminals and subsequent glutamate allosteric potentiation of adjacent GABA receptors plays important roles in some physiological and pathological processes.^23^, ^24^ Under certain conditions, such as increased synaptic activities during the production of certain forms of synaptic plasticity or following ischemic brain insults, extracellular glutamate concentrations can reach levels close to or even above the EC50, which may lead to glutamate allosteric potentiation of the adjacent GABA_A_Rs.^25^ This can result in increased GABA_A_R‐mediated neuronal inhibition and counteract glutamate receptor‐mediated overexcitation. The relatively high EC50 for the glutamate‐mediated allosteric potentiation of GABA_A_Rs is an important feature that ensures glutamate functions as an excitatory transmitter mediating synaptic transmissions at the vast majority of excitatory synapses under most physiological conditions. However, under conditions of overexcitation of glutamatergic neurons and/or compromised glutamate uptake mechanisms, the cross‐talk between glutamate and GABA can potentially bear physiological and pathological significance. Therefore, understanding the mechanisms underlying the co‐release of glutamate and GABA and their functional implications is crucial for the development of effective treatments for various neurological disorders.

The GABA_A_R is a ligand‐gated heteropentameric chloride ion channel that is assembled from families of subunits, including α1‐6, β1‐3, γ1‐3, δ, ε, θ, π, and ρ1‐3.^6^, ^7^, ^8^ The majority of the native GABA_A_Rs are formed by the assembly of two α subunits, two β subunits and one γ subunit with two GABA binding sites.^10^ In particular, β3 subunit‐containing GABA_A_R is broadly expressed in the brain, including cerebral cortex, hippocampus, and hypothalamus.^26^, ^27^, ^28^ Therefore, the β3‐containing GABA_A_Rs have been shown to have important roles in several pathophysiologic processes, such as epilepsy,^29^, ^30^ Angelman syndrome and Prader‐Willi syndrome.^31^, ^32^ Consistent with the involvement of the critical glutamic residue E182 in β3 (E182 and E181 for β1 and β2, respectively), we identified in recombinant GABA_A_Rs,^11^ the β3_E182G_ KI mice, had no effect on fertility but grew slightly slower, exhibit impaired glutamate‐GABA_A_R crosstalk, as evidenced by the significantly reduced potentiation of GABA_A_R‐mediated responses by exogenously applied glutamate and glutamate‐like ligand AP5, in comparison with that in the wildtype counterparts. These results not only provide further evidence supporting the functional operation of the newly identified glutamate‐GABA_A_R cross‐talk in wild‐type animals but also confirm the successful disruption of the cross‐talk in the KI mice.

Neurophysiological and pathological phenotypes observed in these β3‐specific glutamate‐GABA_A_R cross‐talk deficient mice reveal the important physiological and pathological roles of this cross‐talk in vivo. TBS induces much greater GABA_A_R‐mediated IPSCs in WT mice than in β3_E182G_ KI mice, which suggests the stimulation causes neurons to fire at high frequency and results in an increased concentration of glutamate endogenously being released from their axonal terminals. The fact that the potentiation is absent in the glutamate‐GABA_A_R cross‐talk compromised β3_E182G_ KI mice strongly suggests that the theta‐burst stimulation‐induced enhancement of GABA_A_R function is indeed mediated by the glutamate‐GABA_A_R cross‐talk. Since theta‐burst is one of the common neuronal rhythms that occur in the brain under physiological conditions, our results strongly argue for the functional engagement of the cross‐talk as a homeostatic feedback mechanism under physiological conditions. Knocking in the single E182G at the β3 subunit appeared to have no significant impact on the basal function of synaptic GABAARs, including sIPSC and mIPSC. Notably, the tonic inhibitions, which are essential for maintaining neuronal excitability, are mainly mediated by these extrasynaptic. Further identification of the tonic inhibition would be of great significance for GABA_A_Rs' basal function in the future.

Compromising the function of this homeostatic feedback is expected to lead to neuronal overexcitation phenotypes. This is supported by two lines of evidence presented in the current study. First, in comparison with the wild type counterparts, the KI mice showed a significant decrease in their thresholds to nociceptive stimulations to the limbs (Figure 4A,B). While GABA_A_Rs in the central nucleus of the amygdala have an important role in pain control,^33^, ^34^ the findings strongly support that glutamate allosteric potentiation of GABA_A_R is an essential process in GABA_A_R mediated pain controls. Second, intraperitoneal injection of KA at the same concentration induced more severe seizures with increased severity and shortened latency in the KI mice than in the wildtype controls (Figure 4C,D). Meanwhile β3_E182G_ KI mice had more severe seizures with elevated severity and reduced latency than the β2_E181G_ KI mice, indicating that the single E182G mutation at the β3 subunit appears to increase neuronal network excitability phenotype compared to E181G mutation in the β2 subunit. These results not only provide further evidence for the widely accepted roles of GABA_A_Rs in epileptogenesis^14^, ^35^ but also land additional support for the physiological and pathological significance of the glutamate‐GABA_A_R cross‐talk in fine‐tuning E/I balance, and thereby controlling neuronal excitability.

Many previous studies have reported that the E/I balance plays critical roles in normal brain functions; accumulating studies are showing that disruption of this balance may cause neuropsychiatric disorders, such as autism.^16^, ^17^, ^18^, ^19^ Consistent with these findings, we observed in the present study that the β3_E182G_ KI mice exhibit increased locomotor activity and impaired social interaction (Figure 5A–F), which are the two major phenotypes associated with autistic spectrum disorders. However, it is interesting to note that the KI mice unexpectedly exhibit reduced anxiety‐like behavioral phenotype (Figure 5G–L) and enhanced hippocampus‐related spatial learning and memory (Figure 3). One possibility is that due to the disrupted glutamate‐GABA_A_R cross‐talk, β3_E182G_ mutation impairs the ability of the GABA_A_R to adaptively increase its function in response to enhanced glutamate transmission. The inability to adaptively increase GABAergic inhibition results in a relative deficit in GABA_A_R‐mediated inhibition, contributing to the reduced anxiety and increased learning ability. Indeed, previous studies have shown that the GABA_A_ receptor antagonist bicuculline is able to improve both spatial learning and working memory, and decrease anxiety in hyperammonemic rats.^13^ Nonetheless, our study provides strong evidence that disruption of this homeostatic feedback mechanism mediated by the glutamate‐GABA_A_R cross‐talk leads to atypical phenotypes of autistic spectrum disorders.

The majority of phenotypes in β3_E182_ mice are hippocampus‐dependent, we therefore overexpressed recombinant GABA_A_Rs containing the WT β3 subunits in hippocampal neurons and examined its ability to rescue the electrophysiological and behavioral phenotypes in the KI mice. As expected, expression of functional WT β3 subunit‐containing GABA_A_Rs can rescue electrophysiological and behavioral phenotypes, further indicating that the impairment of glutamate allosteric potentiation of GABA_A_Rs and behavioral changes in the KI mice are due to the point mutation of residue E182 of β3.

Notwithstanding these contributions, however, there are some limitations. Social and communication deficits are typical symptoms of ASD,^36^ and many studies are showing that suppressed GABAergic inhibition is a common feature of the autistic brain.^37^ Additionally, ASD is often accompanied by other mental diseases, such as hyperactivity and anxiety.^38^ The β3_E182G_ KI mice exhibited hyperactivity in the open field test and elevated plus maze test but did not display anxiety‐related phenotype. Although, the phenotype of β3_E182G_ KI animal model differs somewhat different from the human condition, additional behavioral assessments would have to be conducted to verify the anxiety‐related phenotypes of β3_E182G_ KI in the future. Furthermore, given the high comorbidity of seizure disorders and autism,^37^ although the severity of KA‐induced seizure activity was significantly increased in KI mice, further electroencephalogram (EEG) recordings of β3_E182G_ KI mice with or without KA‐induced seizure to explore more underlying mechanisms in the future.

Taken together, our results provide strong evidence supporting the mechanistic and functional framework of our newly identified glutamate‐GABA_A_R cross‐talk.^11^ This cross‐talk plays a homeostatic role in maintaining a proper balance between glutamate‐mediated neuronal excitation and GABA‐mediated neuronal inhibition under both physiological and pathological conditions. Disruption of this cross‐talk will lead to various phenotypes associated with neuronal hyperexcitability. Characterization of this cross‐talk in detail can be expected to shed more light into mechanisms of how the brain maintains a proper neuronal E/I balance, and how its deficiency contributes to the pathogenesis of certain brain disorders. It may also provide scientific basis upon which new therapeutics can be developed for treating these brain disorders. This previously unrecognized cross‐talk between the two principle transmitter systems in the mammalian brain not only blurs the traditional distinction between excitatory and inhibitory transmitters but also necessitates further investigation into its physiological and/or pathological roles.

## 4 MATERIALS AND METHODS

### 4.1 Animals

Wild‐type (WT) and β3_E182G_ KI mice were kept in a colony room with a temperature‐controlled (21°C) 12‐h light/12‐h dark cycle, food and water were available ad libitum, and all electrophysiological and behavioral assessments were performed during the light cycle at the age of postnatal day 90. All procedures were performed in accordance with the Chongqing Science and Technology Commission guidelines for animal research and approved by the Children's Hospital of Chongqing Medical University Animal Care Committee.

### 4.2 Generation of β3_E182G_ KI mice

The β3_E182G_ KI mice were generated by Shanghai Model Organisms Center, Inc. (Shanghai, China). A double‐nicking approach was applied to reduce potential off‐target mutagenesis. Two potential guide RNA sequences were designed using the Zhang lab software. Cas9 mRNA and guide RNAs were transcribed in vitro with mMESSAGE mMACHINE T7 Ultra Kit (Ambion). Transcribed products were purified with MEGAclearTM Kit (Ambion).

Cas9 mRNA, Guide RNAs (gRNA 1#: AGGCTGTCACTGGCGTGGAA AGG; gRNA 2#: GCTCTACAATGGAGAACTGTGGG) and an ssDNA donor DNA, including β3_E182G_ mutation (sequence: 5′‐ATGACATTGAATTTTACTGGCGTGGCGGGGACAAGGCTGTCACTGGCGTGGAAAGaATCGgGCTgCCACAGTTCTCCATTGTAGAGCACCGTCTGGTCTCCAGGAATGTTGTCTTCGCCA‐3′) were microinjected into fertilized eggs (C57BL/6J) before the eggs were implanted into surrogate C57BL/6J females. Primers 5′‐AAGGGAGAAAGGGAGGATAGAGGA‐3′ (Gabrb3‐F) and 5′‐GCCAAGAATGAAAAGCAACTGAGA‐3′ (Gabrb3‐R) were used for genotyping (producing a 1.8 kb band) of F0 generation. F0 mice with the correct genotypes were used for F1 breeding. Primers Gabrb3‐F and Gabrb3‐R were also used for F1 genotyping. The heterozygous β3_E182G_ mice were backcrossed three times to WT C57BL/6J mice before further experiments, and multiple litters were used for all experiments to eliminate potential off‐target mutations. Homozygous β3_E182G_ mice from F4 to F8 were used for electrophysiological and behavioral assays.

### 4.3 Adeno‐associated virus and microinjection

To express recombinant α1/β3 GABA_A_Rs in vivo, adeno‐associated virus expressing α1 (AAV_α1_) and β3 (AAV_β3_) were constructed by OBiO Technology (Shanghai, China). Titers were 5 × 10^12^ TU/ml. After anesthetization with sodium pentobarbital, mice at the age of 2 months old were mounted on a stereotaxic instrument, and 0.8 μl of AAV_α1_ and AAV_β3_ were co‐microinjected into the dorsal hippocampal CA1 area through a drilled hole (−2.3 mm posterior, ± 2.0 mm lateral and 2.5 mm ventral relative to bregma). One month after AAVs microinjection, the electrophysiological and behavioral tests were performed. For electrophysiological experiment, only the cells that expressed both the α1 and β3 subunits of GARA_A_R were selected for recordings.

### 4.4 Electrophysiology studies

Mice were deeply anesthetized with urethane (1.5 g/kg, i.p.) and transcardially perfused with artificial cerebral spinal fluid (in mM: NaCl 124, KCl 2.8, NaH_2_PO_4_.H_2_O 1.25, CaCl_2_ 2.0, MgSO_4_ 1.2, Na‐vitamin C 0.4, NaHCO_3_ 26, Na‐lactate 2.0, Na‐pyruvate 2.0 and D‐glucose 10.0, pH = 7.4) prior to decapitation as described previously.^39^ Whole‐cell patch clamp recordings were utilized to investigate the properties of evoked IPSCs (eIPSCs) and spontaneous IPSCs (sIPSCs) in pyramidal neurons of the CA1 subregion of the hippocampus. The recordings were performed using an EPC10 patch clamp amplifier controlled by Patchmaster software. For voltage‐clamp experiments, the internal solution contained 140 mM CsCl, 10 mM HEPES, 4 mM K‐ATP, 0.5 mM EGTA, 0.15 mM CaCl_2_, 4.25 mM MgCl_2_, with pH adjusted to 7.2 with CsOH and osmolarity set to 290–300 mOsm with sucrose. The extracellular solution consisted of 140 mM NaCl, 5.4 mM KCl, 10 mM HEPES, 1.0 mM MgCl_2_, 1.3 mM CaCl_2_, and 20 mM glucose, with pH adjusted to 7.4 using NaOH and osmolarity set to 305–315 mOsm using glucose. To induce eIPSCs, a concentric stimulation electrode was placed at the Schaffer collateral pathway and the stimulation intensity was set to 50% of the maximal eIPSC response. CNQX (20 μM) was added to the bath to block AMPA receptors. After obtaining a stable baseline, D‐AP5 (50 μM) or L‐Glu (50 μM) was added to the recording solution to measure the potentiation of GABA_A_R‐mediated inhibitory currents. Synaptic TBS was delivered, consisting of 5 trains of 4 pulses at 100 Hz, with an inter‐train interval of 200 ms. For recordings of mIPSCs (TTX (0.5 μM) was used to block voltage‐gated Na^+^ channels) and sIPSCs, CNQX (20 μM) and D‐AP5 (50 μM) were added to the recording solution to block action potential and glutamatergic transmission, respectively.

GABA reversal potential (E_GABA_) recordings were performed using a perforated patch‐clamp technique. Recording pipettes (5–6 MΩ) filled with the intracellular solution that contained (mM): K‐Gluconate 136.5, KCl 17.5, NaCl 9, EGTA 0.2, HEPES 10, MgCl2 1, pH 7.2; osmolarity, 285 mOsm. Gramicidin at a concentration of 50 μg/ml was used as the pore‐forming agent for perforated recordings. Around 20–40 min after giga seal formation, the access resistance slowly dropped and stabilized at ∼40 MΩ. Holding potentials were stepped from −110 to −10 mV. At each holding potential step, GABA (100 μM, 50 ms) was perfused directly onto CA1 pyramidal neurons through a pipette (2–3 mm tip) using a VC3 perfusion delivery system (Plexon). During recording, CNQX (10 μM), D‐AP5 (50 μM) and TTX (0.5 μM) were added in extracellular solution to block AMPA receptors and NMDA receptors and voltage‐gated sodium channels, respectively. All experiments were performed at room temperature.

### 4.5 Pain threshold tests

The mechanical withdrawal thresholds were ascertained to assess mechanical hyperalgesia, as described previously.^33^ Each animal was put into an 8 × 9 × 8 cm clear plastic cage with wire mesh to allow the filament to be inserted from below. Prior to testing, the filament was applied to the left hind paw's plantar surface and allowed to acclimatize for at least 10 min. Three measurements were carried out, each at 30‐s intervals. The mechanical withdrawal threshold was based on the average value.

A hot plate test (Stoelting, Wood Dale, IL, USA) was used to measure thermal hyperalgesia. Mice were individually put on a hot plate heated to 55 ± 0.5°C. Withdrawal of the paw from the thermal stimulus was measured by the latency from the onset to the time of hind paw jumping or licking. The test has a 90‐s cutoff time to prevent harm and damage. Double‐blind methods were used to evaluate both pain‐related behavioral tests.

### 4.6 KA‐induced seizures

A 20 mg/ml concentration of KA was dissolved in sterile saline. Intraperitoneal injections (i.p.) of KA (20 mg/kg) or saline in the same volume as the vehicle control were used to cause seizures.^40^ Thirty minutes prior to the injection of KA, diazepam (20 mg/kg, s.c., procured from Children's Hospital of Chongqing Medical University) was administered. A trained observer who was unaware of the mice's genotype or therapy evaluated seizure activity every 15 min for 2 h using the scale as described previously.^40^

### 4.7 Social interaction test

Social interaction was evaluated by using a three‐chamber apparatus as described previously.^41^ Briefly, on day 1, WT and KI mice were left free to explore the chamber for 10 min. Twenty‐four hours later, the first test (test 1) was performed. During test 1, a toy (T) or a stranger mouse (SM) was housed in each side chamber of an identical round cage, while the middle chamber was left unoccupied. A C57BL/6J mouse of the same sex, similar age, and without prior contact with the test animal was utilized as a “stranger.” The test mouse was placed in the central chamber and allowed to explore all chambers for 10 min. Immediately after test 1, the test mouse was co‐housed with the paired mouse during test 1 for 24 h to familiarize with it (FM). On day 3, the second test (test 2) was performed. During test 2, the toy was replaced by another age‐ and gender‐matched stranger mouse (SM). The test mouse was placed in the central chamber and allowed to explore all chambers for another 10 min. The entries and time spent in sniffing in the “interaction zone” (3 cm around the cage) was monitored by ANY‐Maze Video Tracking System (Stoelting, Wood Dale, IL, USA). The social index (SI) was calculated by using the equation: SI on test 1 = number or time spent on M / number or time spent on T; SI on test 2 = number or time spent on SM / number or time spent on FM.

### 4.8 Open field test

Mice were individually placed in a 40 × 40 × 40 cm open field arena for 10 min. ANY‐Maze Video Tracking System (Stoelting, Wood Dale, IL, USA) was used to record and analyze the distance traveled, the number of times it entered the central area and the time spent in the central area.

### 4.9 Elevated plus maze

The elevated plus maze apparatus consisted of two opposing open arms (30 × 5 × 0.3 cm) and two opposing closed arms (30 × 5 × 15 cm). The maze was placed at a height of 75 cm from the floor. Mice were individually left in the center of maze facing the open arm during 10 min. The time spent in each arm and number of entries into each arm were recorded and analyzed by ANY‐Maze Video Tracking System (Stoelting, Wood Dale, IL, USA).

### 4.10 Novel object recognition test

Mice were placed in the 40 × 40 cm open box for 5 min for adaption 24 h before the test. Mice were placed in the box for 5 min to explore two identical objects on the test day, after which they were returned to their food cages. Two hours later, the mice were returned to the box, where one of the objects was replaced by a novel object. The animals were allowed to explore both objects for another 5 min. The experimenter recorded the occurrence of head dips to the objects in a double‐blinded fashion. The recognition index (RI) was calculated by using the equation: RI = number or time spent on novel object/total number or time spent on both objects.

### 4.11 Barnes maze test

The Barnes maze was used to study hippocampus‐based spatial learning and memory.

The apparatus is made of a 1.2‐m diameter white circular platform with 18 holes (5‐cm diameter) around the edge, one of which has an escape box underneath. To record the latency and number of errors made while trying to find the escape box, a charge coupled device (CCD) camera was positioned above the maze center. Video outputs were then digitalized using an ANY‐Maze Video Tracking System (Stoelting, USA). Animals were given 3 min to acclimatize to the maze 24 h before spatial training. Then, for the following 6 days, the animals underwent two trials per day of training in a spatial learning task with a 15‐min interval break. If the mouse was unable to find the box, or if it did find the box but failed to enter it within 5 min, it would be gently guided to the escape box and remained there for 60 s before being returned to its home cage. Twenty‐four hours following the last training trial, mice were subjected to a 5‐min probing test while the escape box was blocked. The correct ratio was calculated by using the equation as described previously^42^: % correct ratio = number of finding the escape box/total number of reaching all holes × 100%.

### 4.12 Data analysis

Values were expressed as mean ± SEM (*n* = number of experiments). The data of the Barnes maze training and seizure class were analyzed by a two‐way between/within‐subjects factorial analysis of variance (ANOVA), with genotype as the between‐subjects factor and training session (time) as the within‐subjects factor. All significant main effects and interactions were further analyzed using Turkey's comparisons. Other data were analyzed by a two‐tailed student's *t*‐test. All statistical analyses were performed using SPSS 22.0, and GraphPad Prism8.0.2. Statistical significance was set at *p* < 0.05.

## AUTHOR CONTRIBUTIONS

YD, YTW, and ZD conceived the study and wrote the manuscript. YD, MW, and QT performed phenotypical analysis and behavioral studies. JL, YP, YW, and DCW performed electrophysiological studies. All authors have read and approved the article.

## CONFLICT OF INTEREST STATEMENT

The authors declare no conflicts of interest.

## ETHICS STATEMENT

Animal ethics approval is approved by the Animal Ethics Committee of Children's Hospital of Chongqing Medical University (approval number: CHCMU‐IACUC20210114017).

## Supporting information

Supporting Information

## Data Availability

The datasets generated during and/or analyzed during the current study are available from the corresponding author upon reasonable request.
