# Supplementary material for: Genetic inhibition of glutamate allosteric potentiation of GABAARs in mice results in hyperexcitability, leading to neurobehavioral abnormalities
Source: MedComm (2020). 2023 Apr 24;4(3):e235. doi: 10.1002/mco2.235 (PMC10123808; doi:10.1002/mco2.235)
Supplement: Supplementary file 1 — Supporting Information [file MCO2-4-e235-s001.docx]

**Title:** Genetic inhibition of glutamate allosteric potentiation of GABA_A_Rs in mice results in hyperexcitability, leading to neurobehavioral abnormalities

**Running title:** Glu-GABA_A_R crosstalk ensures brain function

**Author names and affiliations:**

Yehong Du^1#*^, Junjie Li^1#^, Maoju Wang^1^, Qiuyun Tian^1^, Yayan Pang^1^, Ya Wen^2^, Dongchuan Wu^3^, Yu Tian Wang^2^, Zhifang Dong^1,4*^

^1^Growth, Development, and Mental Health of Children and Adolescence Center, Pediatric Research Institute, Ministry of Education Key Laboratory of Child Development and Disorders, National Clinical Research Center for Child Health and Disorders, China International Science and Technology Cooperation Base of Child Development and Critical Disorders, Chongqing Key Laboratory of Translational Medical Research in Cognitive Development and Learning and Memory Disorders, Children’s Hospital of Chongqing Medical University, Chongqing 400014, China

^2^Brain Research Centre and Department of Medicine, Vancouver Coastal Health Research Institute, University of British Columbia, Vancouver, BC V6T 2B5, Canada

^3^Translational Medicine Research Center, China Medical University Hospital, Graduate Institutes of Biomedical Sciences, Taichung, Taiwan, China

^4^Institute for Brain Science and Disease of Chongqing Medical University, Chongqing 400016, China

^#^YD and JL contributed equally

^*^Correspondence to: [zfdong@cqmu.edu.cn](mailto:zfdong@cqmu.edu.cn) (ZD) or [dudu0000807@126.com](mailto:dudu0000807@126.com) (YD)

**Supplementary figures and figure legends**

**
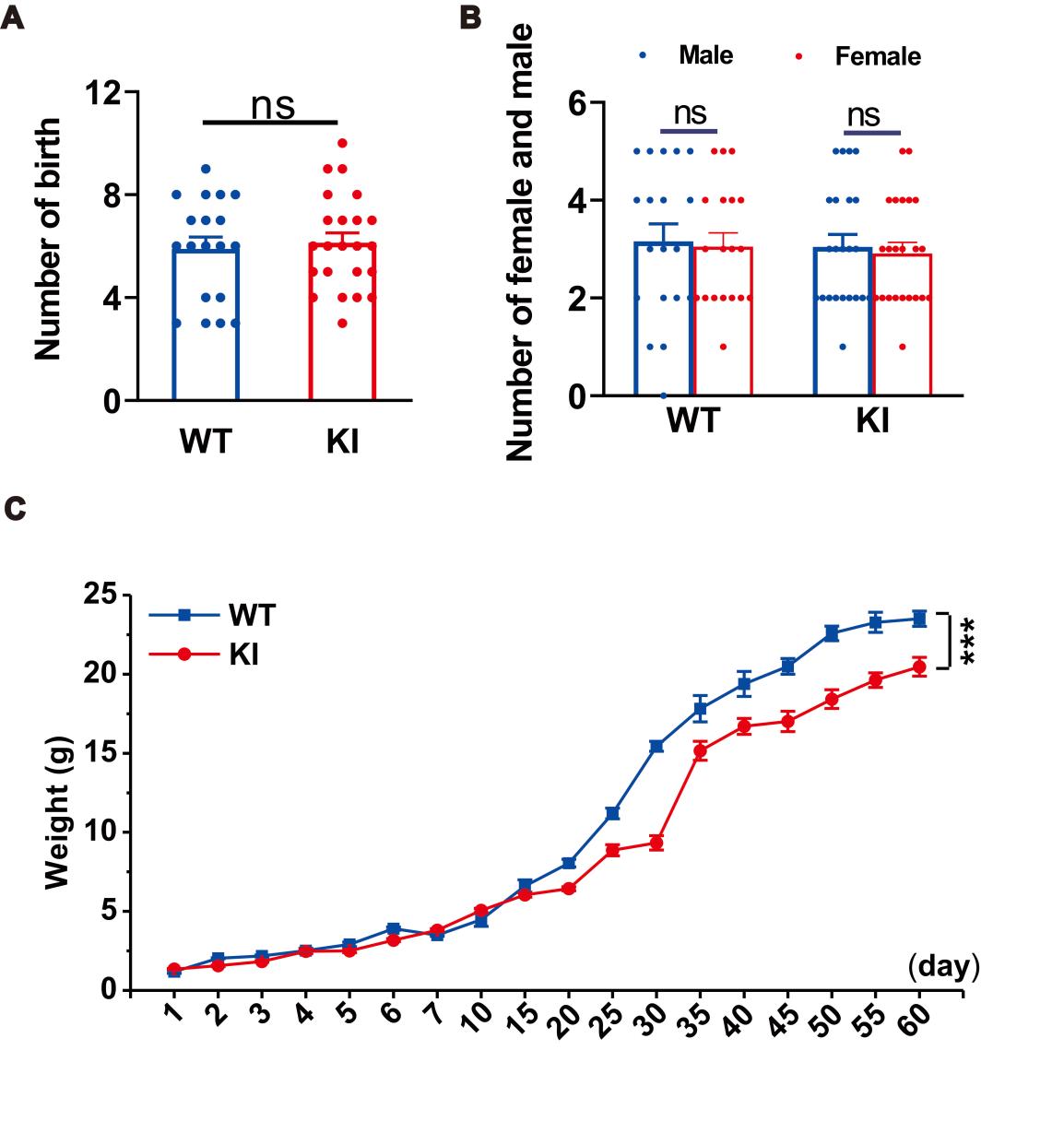
**

**Figure S1. KI mice does not alter the growth curve, number of births and female/male ratio. A**. The number of births shows no significant difference in both groups (WT: n = 19; KI: n = 23). **B**. The number of female and male shows no significant difference in both groups (WT: n = 19; KI: n = 23). **C**. The growth curve shows that KI mice grow slightly slower (~10% lighter than WT mice) (WT: n = 13; KI: n = 21, p < 0.001). Data are expressed as mean ± SEM; ***p < 0.001.

**
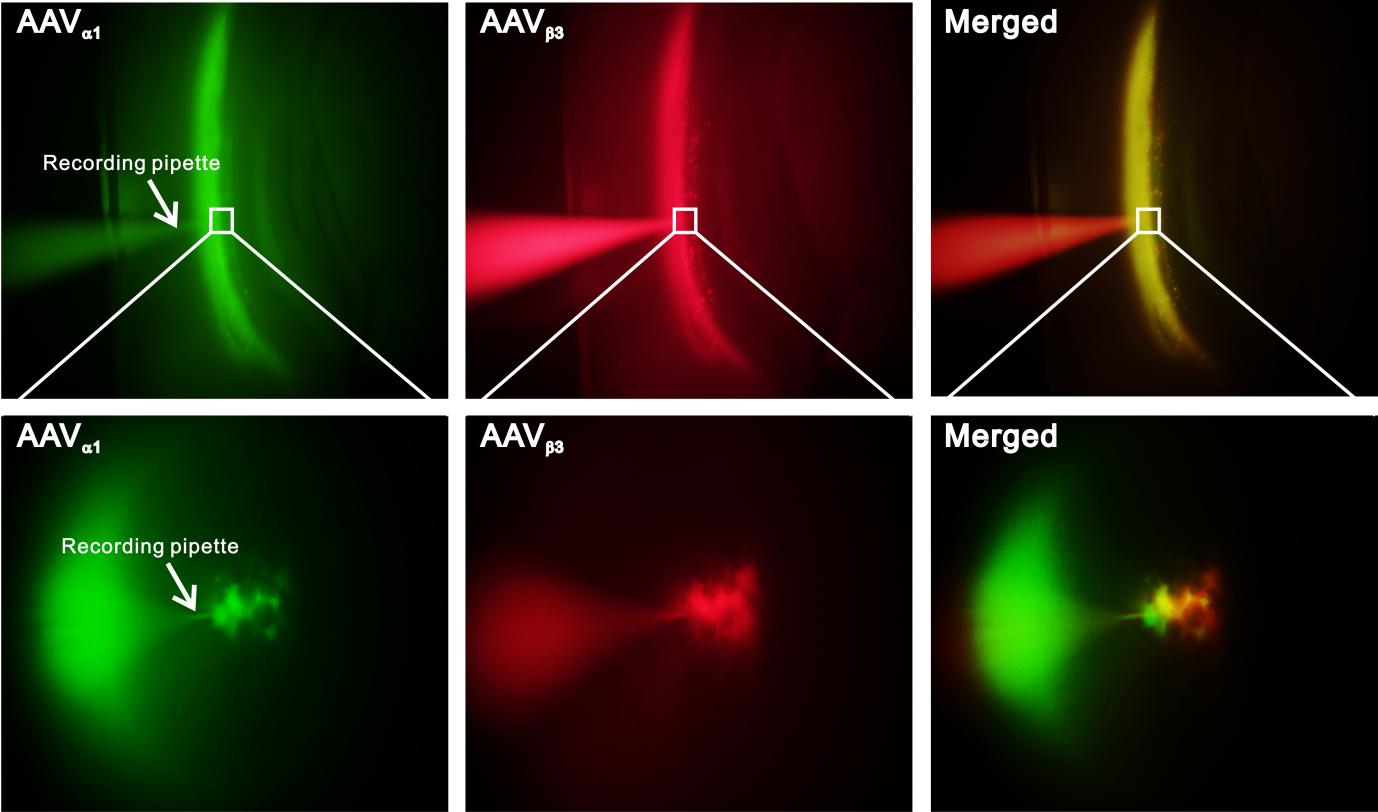
**

**Figure S2. Co-infected of AAV_α1_ and AAV_β3_ into the hippocampal CA1 neurons**. Electrophysiological recordings are performed in the hippocampal CA1 pyramidal neurons that were co-expressed of fluorescently identified recombinant GABA_A_Rs containing both α1 (Green) and β3 (Red).
